# Multiple neck operations in a patient with severe motor tics because of Tourette’s syndrome: a case report

**DOI:** 10.1186/1752-1947-6-223

**Published:** 2012-07-30

**Authors:** Tomohiro Miyashita, Masashi Yamazaki, Akihiko Okawa, Minori Yoneda, Atsuomi Aiba, Masao Koda, Kazuhisa Takahashi

**Affiliations:** 1 Spine Section, Department of Orthopaedic Surgery, Chiba University Graduate School of Medicine, Chiba, Japan

## Abstract

**Introduction:**

In patients with Tourette’s syndrome who have severe motor tics, involuntary neck movements can enhance degenerative changes in the cervical spine, occasionally causing myelopathy. There have been a limited number of reports on surgical treatment for cervical myelopathy caused by Tourette’s syndrome, and a consensus for surgical treatment has not been fully established. To the best of our knowledge, this is the first report that describes a case of cervical myelopathy in a patient with Tourette’s syndrome with severe motor tics who has undergone multiple surgeries of the cervical spine.

**Case presentation:**

A 44-year-old Asian man with severe motor tics due to Tourette’s syndrome presented with cervical myelopathy. Previously, he had undergone an anterior discectomy and spinal fusion with ceramics at the C3-C4 and C5-C6 levels, but required further surgery because of displacement of the ceramics. After the second operation, he developed compression myelopathy at the sandwiched (C4-C5) disc level, and had to undergo a C4-C5 anterior discectomy and spinal fusion, which was unsuccessful.

As a salvage operation, we performed a C3-C7 decompression and spinal fusion from both the anterior and posterior approaches. By thorough postoperative external immobilization of his neck, our patient’s spinal fusion was successful and his neurological improvements were maintained for more than 10 years.

**Conclusions:**

Patients with Tourette’s syndrome with cervical myelopathy are at risk of having multiple neck operations to correct their symptoms. Postoperative immobilization and the correct selection of surgical procedure are quite important for successful spinal fusion and for avoiding complications at adjacent levels in these patients.

## Introduction

Tourette’s syndrome is a complex, childhood-onset, neurobehavioral disorder characterized by chronic motor and phonic tics [1]. In patients with Tourette’s syndrome and severe motor tics, involuntary neck movements can enhance the development of cervical spondylosis and/or disc herniation, resulting in cervical myelopathy [2-9]. Surgical treatment is indicated for patients with Tourette’s syndrome who develop cervical myelopathy. However, to the best of our knowledge, only seven cases of surgical treatment have been reported: four cases of decompression surgery [2-5] and three cases of decompression with spinal fusion [6,9] (Table 1). In these patients, when postoperative management of their involuntary neck movements was inadequate, the surgical outcomes were not necessarily sufficient. Adler *et al*. reported the case of a patient in whom the fusion was broken after surgery [6], Krauss and Jankovic reported a patient with late neurological deterioration that occurred several years after surgery [2] and Dobbs and Berger reported the case of a patient whose symptoms worsened just 10 weeks after surgery [5]. The other four cases were not followed-up over the long term [3,4,9]. 

**Table 1:** Reports of surgery for cervical myelopathy associated with Tourette’s syndrome

**Age**	**Sex**	**Operation**	**External fixation**	**Follow-up**	**Outcome**	**Reference**
27	M	C6/7 discectomy	ND	2 months	Improved	[3]
38	M	C3-T2 laminectomy + C6-7 ASF + C7-T1 PF	Cervical traction	ND	Fusion breaking	[6]
23	M	C3-5 laminectomy	ND	29 years	Recurred	[2]
15	M	C3-7 laminoplasty	ND	ND	Improved	[4]
25	M	C5/6 discectomy	ND	3 years	Recurred	[5]
52	M	C2-5 laminectomy + LMS	Halo vest	6 months	Improved	[9]
52	M	C3-7 laminectomy + LMS	ND	7 months	Improved	[9]

*ASF* anterior spinal fusion; *LMS* lateral mass screw fixation; *M* male; *ND* not described; *PF* posterior fusion.

We report a case of cervical spondylotic myelopathy in a patient with severe motor tics because of Tourette’s syndrome. Our patient had undergone three surgeries for myelopathy prior to visiting our clinic. We performed multisegment spinal decompression and fusion from both the anterior and posterior approaches for this patient. With postoperative external immobilization of his neck, our patient’s spinal fusion was successful and his neurological improvements were maintained for more than 10 years.

### Case presentation

A 44-year-old Asian man was admitted to our hospital complaining of sensory disturbances in his four extremities and trunk, as well as weakness of his upper extremities, and clumsy hand and gait disturbances. He had an unremarkable childhood, but developed tics and was diagnosed with Tourette’s syndrome at age 24. At the age of 33, he noticed numbness in his right hand, which gradually worsened. He visited a hospital for his symptoms and was diagnosed with compression myelopathy at the C3-C4 and C5-C6 levels. At the age of 38, he underwent anterior discectomy and spinal fusion with ceramics at the C3-C4 and C5-C6 levels. Two weeks later, our patient underwent an iliac bone graft at the C3-C4 and C5-C6 levels because of displacement of the ceramics. Postoperatively, he was immobilized in a halo vest for 2.5 months. After the second surgery, his spinal fusion was successful and his neurological symptoms improved. However, 5.5 years after the second surgery, he began experiencing numbness in his left leg after sustaining a fall. Subsequent to that, he presented with weakness of his right leg. He was once again diagnosed with compression myelopathy that developed at the sandwiched (C4-C5) disc level, and he underwent a C4-C5 anterior discectomy and spinal fusion at the same hospital. After the third surgery, his symptoms were temporarily relieved, but recurred 3.5 months later (one month prior to his visit to our clinic).

**Figure 1:**
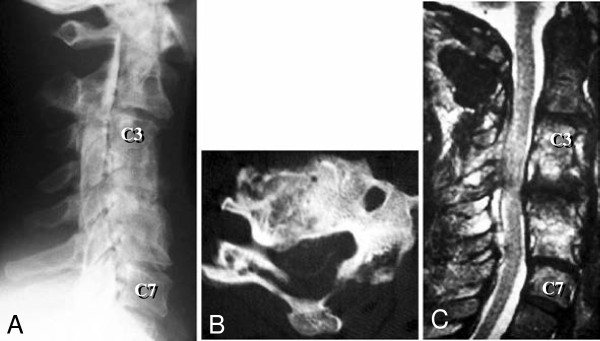
**Three images obtained upon admission to our hospital (4.5 months after the patient’s third surgery).** (**A**) A lateral view myelogram showing anterior compression of the dural tube at the C4-C5 level. (**B**) An axial computed tomographic myelogram showing that the spinal cord is compressed from the anterior and posterior directions at the C4-C5 level. (**C**) A T2-weighted midsagittal magnetic resonance image showing spinal canal stenosis at the C4-C5 level due to pseudoarthrosis of the C4-C5 fusion.

On admission to our hospital, our patient was able to walk independently but his gait was slow and unsteady. His muscle strength in the lower limbs was normal, but his deltoid, biceps and triceps muscles were somewhat weak bilaterally. His pinprick sensation was reduced up to the level of the C6 dermatome. Hyperreflexia was present at his bilateral patellar and Achilles tendons, and patellar and ankle clonus were transiently positive bilaterally. His Babinski sign was present, and his bladder function was slightly disturbed. A myelogram showed pseudoarthrosis of the C4-C5 fusion and anterior compression of the dural tube at the C4-C5 level (Figure 1A). A computed tomographic (CT) myelogram and a magnetic resonance (MR) image showed spinal canal stenosis at the C4-C5 level (Figure 1B, C).

**Figure 2:**
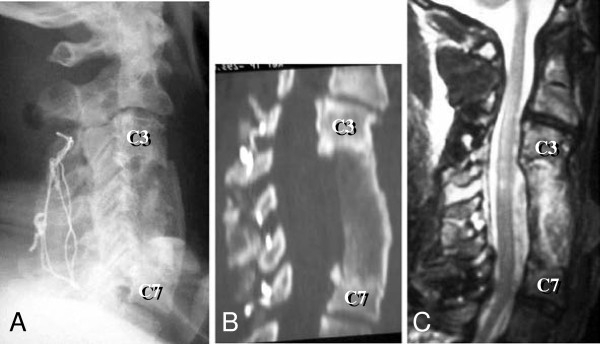
**Three images following C3-C7 decompression and spinal fusion from both the anterior and posterior approaches.** (**A**) A lateral view postoperative radiogram taken immediately after the fourth surgery showing good alignment of the cervical spine. (**B**) A midsagittal computed tomographic reconstruction taken three months after the fourth surgery showing a mature fusion mass at C3-C7. (**C**) A T2-weighted midsagittal magnetic resonance image taken one year after the fourth surgery showing appropriate decompression of the spinal cord.

Our patient was prepared for surgical treatment, which was initially planned as a posterior fusion and an anterior decompression and interbody fusion with electrophysiological monitoring of spinal cord activity. We first performed posterior fusion of C3-C7 using Bohlman’s triple-wire technique, which was uneventful. Next, we performed anterior corpectomy of C4, C5 and C6, and spinal fusion at C3-C7 with a strut graft using autologous iliac bone (Figure 2A).

**Figure 3:**
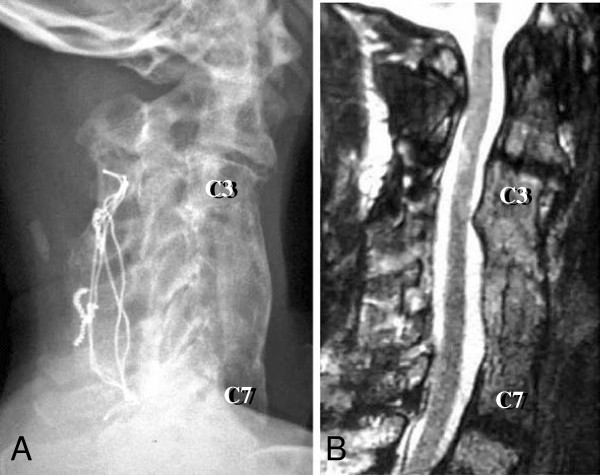
**Two images taken six years after the fourth surgery.** (**A**) A lateral view cervical radiogram showing successful interbody fusion. (**B**) A T2-weighted midsagittal magnetic resonance image showing mild disc degeneration at the adjacent C2-C3 and C7-T1 levels, but no spinal cord compression.

Postoperatively, our patient was fitted with a halo vest for the first three months and a cervical collar for another three months. After application of the halo vest his tics worsened, possibly due to the discomfort he felt by the immobilization of his neck, and the pins became loose several times. We had to regulate the torque of the pins every day, and replaced them four times. We treated his tics with haloperidol and his involuntary neck movements were slightly reduced. However, we could not increase the dose because of drowsiness. Botulinum toxin was not used for treating such a patient in our hospital at that time. A midsagittal CT reconstruction three months after surgery showed a mature fusion mass (Figure 2B). A T2-weighted MR image one year after surgery revealed that his spinal cord was well decompressed (Figure 2C). A lateral cervical radiogram taken six years after surgery showed successful interbody fusion, and a T2-weighted MR image taken at the same time detected only slight degeneration of the adjacent discs of the fusion site (Figure 3).

Our patient’s neurological deficits gradually recovered after surgery. His clumsy hand movements disappeared four months after surgery, and at his last follow-up examination, 10 years after the surgery, he was able to perform all activities of daily living.

## Discussion

Tourette’s syndrome is an inherited developmental disorder of synaptic neurotransmission resulting in the disinhibition of the cortico-striatal-thalamic-cortical circuitry [10]. Although one diagnostic criterion is onset before the age of 21, the disorder manifests by the age of 11 in 96% of patients, typically beginning between three and eight years of age. Secondary neurologic deficits such as compressive cervical myelopathy often occur in these patients as a result of violent head and neck tics.

Many reports advocate surgical treatment for cervical myelopathy in patients with athetoid cerebral palsy [9,11-18], and surgical procedures and postoperative strategies have been established. However, there have been a limited number of reports on cervical myelopathy in patients with Tourette’s syndrome, and a consensus for treatment has not been fully determined [2-9].

Anterior and/or posterior decompression with spinal fusion to surgically treat patients with athetoid cerebral palsy has been reported, and the long-term surgical outcomes in these patients were not unsatisfactory. Postoperative immobilization of the neck plays an important role in the success of the surgery. For this purpose, external fixation with a halo vest is a representative procedure. Regulation of involuntary neck motion is also an important factor for obtaining good surgical outcomes. Problems derived from involuntary neck motion include pseudoarthrosis of the fusion site [11,13,17], graft displacement [18], halo pins loosening [9,16] and degenerative changes at a level adjacent to the fusion site [13,14]. In some studies, botulinum toxin has been used for immobilization [9,16]. A previous report on patients with Tourette’s syndrome determined that administration of botulinum toxin had a positive effect on postoperative immobilization of the neck and healing of the fusion [6].

In our case, the ceramic displacement after the first operation and fusion failure after the third operation were likely a result of insufficient postoperative immobilization of the neck. It was inevitable that rigid internal fixation was not used for this patient at that time, when most spinal surgeons had little information about the cervical myelopathy of Tourette’s syndrome and the current cervical spinal surgical techniques did not exist. As mentioned above, there have been reports of graft displacement [18] and fusion failure [11,13,17] in patients with athetoid cerebral palsy. The spinal canal stenosis at the C4-C5 level in this case seemed to be derived from the skipped C3-C4 and C5-C6 fusion of the first operation. After such skipped fusion, degenerative changes of the sandwiched disc may rapidly occur. Shinomiya *et al*. reported that, after anterior spinal fusion in some patients, disc degeneration developed at the adjacent levels, which required the patients to undergo further operations [19]. In the present case, stress on the intervertebral discs seemed to have been increased because of the involuntary movements. This considerably increased the risk that led to the development of spinal canal stenosis at C4-C5 after the skipped C3-C4 and C5-C6 fusion.

In the present case, we performed a multilevel decompression and spinal fusion from both the anterior and posterior approaches as a salvage operation. After surgery, the fusion proceeded well and our patient’s neurological deficits were improved. Hilibrand *et al*. reported that disc degeneration frequently occurred after spinal fusion at the C5-C6 and C6-C7 levels and caused late-onset neurological deterioration [20]. In the present case, in addition to the C4-C5 level, we preventively added the C6-C7 fusion to remove the possibility of postoperative neurological deterioration deriving from disc degeneration at the adjacent levels. Six years after the fourth surgery, slight disc degeneration at the adjacent C2-C3 and C7-T1 levels of the fusion site was detected in an MR image (Figure 3B). We will continue follow-up with special attention to these levels.

Previous studies have suggested that decompression surgeries, such as discectomy, laminectomy and laminoplasty, for patients with cervical myelopathy with severe motor tics are not reliable because of late instability and recurrence of symptoms [2,5,6]. Similarly, in patients with athetoid cerebral palsy, laminectomy is thought to be contraindicated [13]. Onari *et al*. analyzed 20 patients with cervical spondylotic myelopathy and athetoid cerebral palsy, with a mean follow-up of 8.7 years [15]. They reported that combined anterior-posterior fusion could effectively improve neurological function. They performed posterior fixation with wave-shaped rods and did not use postoperative rigid external fixation. Wong *et al*. reported that laminectomy with lateral mass fixation prevented the development of postoperative kyphotic deformity, and produced better surgical outcomes [9]. Our experience with this case suggests that patients with Tourette’s syndrome with cervical myelopathy are at risk of having multiple neck operations to correct their symptoms, and postoperative immobilization and the correct selection of surgical procedure are quite important for successful spinal fusion and for avoiding repeated inevitable failures and complications at adjacent levels. For patients with Tourette’s syndrome and severe motor tics, we should follow the surgical procedures for cervical myelopathy in patients with athetoid cerebral palsy.

The surgical procedure that we performed for this patient more than 10 years ago is certainly outdated and we now perform operations using current techniques, including the use of pedicle screws or lateral mass screws, which can obtain more rigid fixation. We also think that there is now no need for a halo vest as the current rigid fixation can obtain a successful spinal fusion. However, we believe that our case provides valuable information for understanding and successfully treating the cervical myelopathy of patients with Tourette’s syndrome, even when using current techniques.

## Conclusions

Patients with Tourette’s syndrome with cervical myelopathy are at risk of having multiple neck operations to correct their symptoms. Postoperative immobilization and correct selection of a surgical procedure are quite important for successful spinal fusion and for avoiding repeated inevitable failures and complications at adjacent levels in these patients.

## Consent

Written informed consent was obtained from the patient for publication of this case report and any accompanying images. A copy of the written consent is available for review by the Editor-in-Chief of this journal.

## Competing interests

The authors declare that they have no competing interests.

## Authors’ contributions

TM is the corresponding author, and assisted with the operation and wrote the manuscript. MYa conducted the operation, followed-up our patient and has contributed in conception and design and revising the manuscript. MYo, AA and MK assisted with the operation. AO and KT contributed to revising the manuscript. All authors read and approved the final manuscript.

## Authors’ information

TM is the Director of the Spine Center, Matsudo City Hospital, Chiba, Japan.

MYa is an Associate Professor, Department of Orthopaedic Surgery, Chiba University Graduate School of Medicine, Chiba, Japan.

AO is an Assistant Professor, Department of Orthopaedic Surgery, Chiba University Graduate School of Medicine, Chiba, Japan.

MYo is Chief Surgeon, Department of Orthopaedic Surgery, Chiba Prefectural Sawara Hospital, Chiba, Japan.

AA is Chief Surgeon, Department of Orthopaedic Surgery, Numazu City Hospital, Shizuoka, Japan.

MK is the Director, Department of Orthopaedic Surgery, Chiba Aoba Municipal Hospital, Chiba, Japan.

KT is Professor and Chairman, Department of Orthopaedic Surgery, Chiba University Graduate School of Medicine, Chiba, Japan.
